# Incidence Trajectories of Psychiatric Disorders After Assault, Injury, and Bereavement

**DOI:** 10.1001/jamapsychiatry.2023.5156

**Published:** 2024-01-17

**Authors:** Yufeng Chen, Qing Shen, Paul Lichtenstein, Jaimie L. Gradus, Filip K. Arnberg, Henrik Larsson, Brian M. D’Onofrio, Fang Fang, Huan Song, Unnur A. Valdimarsdottir

**Affiliations:** 1 Unit of Integrative Epidemiology, Institute of Environmental Medicine, Karolinska Institutet, Stockholm, Sweden; 2 Centre of Public Health Sciences, Faculty of Medicine, University of Iceland, Reykjavík, Iceland; 3 Clinical Research Center for Mental Disorders, Shanghai Pudong New Area Mental Health Center, Tongji University School of Medicine, Shanghai, China; 4 Institute for Advanced Study, Tongji University, Shanghai, China; 5 Department of Medical Epidemiology and Biostatistics, Karolinska Institutet; 6 Department of Epidemiology, Boston University School of Public Health, Boston, Massachusetts; 7 Department of Psychiatry, Boston University School of Public Health, Boston, Massachusetts; 8 Department of Clinical Epidemiology, Aarhus University Hospital, Aarhus, Denmark; 9 National Centre for Disaster Psychiatry, Department of Medical Sciences, Uppsala University, Uppsala, Sweden; 10 School of Medical Sciences, Örebro University, Örebro, Sweden; 11 Department of Psychological and Brain Sciences, Indiana University, Bloomington, Indiana; 12 West China Biomedical Big Data Center, West China Hospital, Sichuan University, Chengdu, China; 13 Department of Epidemiology, Harvard T. H. Chan School of Public Health, Boston, Massachusetts

## Abstract

**Importance:**

Traumatic events have been associated with elevated risks of psychiatric disorders, while the contributions of familial factors to these associations remain less clear.

**Objective:**

To determine the contribution of familial factors to long-term incidence trajectories of psychiatric disorders following potentially traumatic events.

**Design, Setting, and Participants:**

This cohort study evaluated 3 separate cohorts of individuals residing in Sweden who were free of previous diagnosed psychiatric disorders when first exposed to assault (n = 49 957), injury (n = 555 314), or bereavement (n = 321 263) from January 1987 to December 2013, together with their unexposed full siblings, and 10 age-, sex-, and birthplace-matched unexposed individuals (per exposed individual). Cohorts were created from the Swedish Total Population Register linked to health and population registers. Data were analyzed from March 2022 to April 2023.

**Exposures:**

Potentially traumatic events, including various types of assault, injuries, and bereavement (death of a child or of a spouse or partner), were ascertained from the Swedish national registers.

**Main Outcomes and Measures:**

Incident psychiatric disorders were ascertained from the Swedish Patient Register. Flexible parametric and Cox models were used to estimate associations of potentially traumatic events with incident psychiatric disorders after multivariable adjustment.

**Results:**

The median (IQR) age at exposure to assault, injury, and bereavement was 22 (18-31), 19 (8-40), and 60 (51-68) years, respectively. During a median (IQR) follow-up of 4.9 (2.2-8.2), 9.1 (4.1-15.6), and 8.1 (3.4-14.8) years, the incidence rates of any psychiatric disorder were 38.1, 13.9, and 9.0 per 1000 person-years for the exposed groups of the 3 cohorts, respectively. Elevated risk of any psychiatric disorder was observed during the first year after exposure to any assault (hazard ratio [HR], 4.55; 95% CI, 4.34-4.77), injury (HR, 3.31; 95% CI,3.23-3.38), or bereavement (HR, 2.81; 95% CI, 2.72-2.91) and thereafter (assault HR, 2.50; 95% CI, 2.43-2.56; injury HR, 1.69; 95% CI, 1.68-1.70; bereavement HR, 1.42; 95% CI, 1.40-1.44). Comparable associations were obtained in sibling comparison (first year: assault HR, 3.70; 95% CI, 3.37-4.05; injury HR, 2.98; 95% CI, 2.85-3.12; bereavement HR, 2.72; 95% CI, 2.54-2.91; thereafter: assault HR, 1.93; 95% CI, 1.84-2.02; injury HR, 1.51; 95% CI, 1.48-1.53; bereavement HR, 1.35; 95% CI, 1.31-1.38). The risk elevation varied somewhat by type of traumatic events and psychiatric disorders, with the greatest HR noted for posttraumatic stress disorder after sexual assault (sibling comparison HR, 4.52; 95% CI, 3.56-5.73 during entire follow-up period).

**Conclusions and Relevance:**

In this study, the long-term risk elevation of psychiatric disorders after potentially traumatic events was largely independent of familial factors. The risk elevation observed immediately after these events motivates early clinical surveillance and mental health services for these vulnerable populations.

## Key Points

**Question:**

Are potentially traumatic events associated with a subsequent risk of psychiatric disorders independent of familial factors?

**Findings:**

In this Swedish nationwide cohort study using a sibling-comparison design to adjust for familial factors, physical and sexual assault (n = 49 957), injury (n = 555 314), and bereavement (n = 321 263) were associated with an increased risk of subsequent psychiatric disorders for more than 2 decades of observation and in particular during the first year following the event.

**Meaning:**

These findings suggest that early clinical surveillance and targeted mental health services may be advisable among individuals who experience assault, injury, or bereavement.

## Teaser

This cohort study evaluates risk of psychiatric disorders following the potentially traumatic events of assault, injury, and bereavement apart from familial factors.

## Introduction

Most individuals experience potentially traumatic events, such as injury or violence, at some point during their lifetime. The recent World Mental Health Survey revealed that 70% of the participants pooled across 24 countries experienced at least 1 lifetime traumatic event (prevalence ranges from 29% to 85% across countries).^1,2^ Depending on the circumstances, bereavement caused by the death of a loved one can also represent a traumatic event experienced by a large proportion of the population.

Mounting evidence suggests a link between various trauma exposures and risks of psychiatric disorders.^2,3,4,5,6,7,8,9,10,11,12^ However, previous studies in this area are often limited to a particular type of trauma or bereavement,^3,8,10,13,14^ events occurring during a specific period (eg, childhood^7,15^), or specific psychiatric disorders (eg, posttraumatic stress disorder [PTSD]),^2,16,17,18,19,20,21,22,23^ without a comprehensive assessment of the incidence trajectories of psychiatric disorders after common traumatic events during the life course.

Importantly, risk-taking behaviors,^24,25,26^ trauma,^27,28^ and psychiatric disorders^29,30,31^ cluster within families, and the genetic contributions to both stressful life events^32,33^ and psychiatric disorders^34,35^ have been estimated to vary from moderate to strong. Hence, the observed associations between potentially traumatic events and psychiatric disorders may be confounded by familial factors. The sibling-controlled design^36^ represents a unique and powerful approach to control for familial factors (eg, genetics, early environment, and lifestyle) in a population-based setting while assessing the long-term temporal pattern of the association between potentially traumatic events and psychiatric disorders.

Therefore, using nationwide registers in Sweden with routinely and prospectively collected information on health care utilization, our study investigated the associations between potentially traumatic events (including assaults, injuries, and bereavement) and the long-term incidence trajectories of psychiatric disorders in the general population, taking into account familial factors through a sibling-controlled design.

## Methods

### Study Design

This study was approved by the regional ethics review board in Stockholm, Sweden. Informed consent is waived by Swedish law when using register-based data.

From the Swedish Total Population Register,^37^ we identified all individuals (N = 8 475 016) born in Sweden from 1932 to 2013 who resided in the country on January 1, 1987, or were born thereafter. Unique personal identification numbers were used to link these individuals to different health and population registers.^38^ We constructed 3 separate cohorts to perform both population and sibling comparisons. We included all individuals exposed to assaults, injuries, or bereavement from January 1987 to December 2013 as exposed groups **(**eFigure 1 in Supplement 1). We excluded individuals with any previous diagnosis of psychiatric disorder or conflicting information (eg, died before exposure or born after exposure), leaving 49 957 individuals exposed to assaults, 555 314 exposed to injuries, and 321 263 exposed to bereavement. Date of exposure was used as the index date for the exposed individuals.

Adhering to the Strengthening the Reporting of Observational Studies in Epidemiology (STROBE) reporting guideline,^39^ in population comparison, we created 3 population cohorts by randomly selecting 10 individuals per exposed individual from the general population who were free of potentially traumatic events and had no psychiatric disorder before index date. Exposed and unexposed individuals were individually matched on birth year, sex, and birthplace (county of birth).

To control for familial factors, we performed a sibling comparison according to guidelines for designing family-based studies.^36^ We identified unexposed full siblings of the exposed individuals through the Swedish Multi-Generation Register, which includes almost complete family information for individuals born since 1932.^40^ The unexposed siblings were included in the analysis if they were free of the studied potentially traumatic events and had no history of psychiatric disorder before the index date. Three sibling cohorts were established separately for assault (exposed individuals = 34 894; unexposed siblings = 56 534), injury (exposed individuals = 388 077; unexposed siblings = 629 434), and bereavement (exposed individuals = 191 034; unexposed siblings = 372 665).

In all cohorts, we followed up with participants from the index date until the first diagnosis of any psychiatric disorder, death, emigration, first exposure to the studied potentially traumatic events (unexposed individuals or unexposed siblings), or end of follow-up (December 31, 2013), whichever occurred first.

### Ascertainment of Potentially Traumatic Events and Psychiatric Disorders

We used the Swedish revisions of the *International Classification of Diseases*, *Ninth Revision *(*ICD-9*), and *Tenth Revision* (*ICD-10*) codes to ascertain first recorded exposure to any assault and any injury with external causes and their subtypes from the Swedish Patient Register, with nationwide coverage of inpatient specialist care since 1987—including outpatient specialist care (>80%) since 2001 with data on both planned and emergency care.^41^ We focused on bereavement due to loss of a child or spouse or partner due to death. We used the Swedish Multi-Generation Register to identify children and, through a common child, spouse or partner of the study participants. We linked the children and spouse or partner to the Causes of Death Register^42^ to identify bereavement events.

We retrieved incident psychiatric disorders during follow-up as outcomes from the Patient Register, using either the primary or secondary diagnosis at discharge. In an additional analysis, we studied suicide attempts and completed suicide as an extreme indication of psychiatric morbidity, using the Patient Register to ascertain suicide attempt and the Causes of Death Register to ascertain completed suicide. Please see the eMethods and eTable 1 in Supplement 1 for details.

### Statistical Analysis

We calculated the crude incidence rates of psychiatric disorders among exposed and unexposed individuals or siblings during follow-up, using the number of cases divided by accumulated person-years at risk. We estimated the time-varying associations between potentially traumatic events and psychiatric disorders using flexible parametric survival models,^43^ contrasting the rates of psychiatric disorders in exposed group with the rates in unexposed individuals or siblings, with time since index date as the underlying time scale. We then separately assessed the associations within and beyond 1 year since the index date using Cox models, with results presented as hazard ratios (HRs) with 95% CIs. The proportional hazards assumption was tested using the Schoenfeld residuals method,^44^ and no violation was noted.

In the population comparison, we stratified all analyses by matching identifiers (sex, birth year, and birthplace) and adjusted for educational level (less than 9 years, 9 to 12 years, more than 12 years, or unknown), family income (top 20%, middle, lowest 20%, or unknown), marital status (single, married or cohabiting, or divorced or widowed), history of severe somatic diseases (yes or no), and family history of psychiatric disorders (yes or no). We conducted subgroup analyses by sex, age in years (age at index date or during follow-up: younger than 18, 18 to 29, 30 to 65, or older than 65), calendar year at index date (1987 to 1996, 1997 to 2006, or 2007 to 2013), family history of psychiatric disorders, and history of severe somatic diseases. In the sibling comparison, we stratified all analyses by family identifiers and adjusted for age at index date, sex, and all above covariates except for family history of psychiatric disorders.

In additional analyses, we first assessed the association between exposure to multiple trauma subtypes and risk of psychiatric disorders and then assessed the risk of suicide attempt or death from suicide in relation to traumatic events. We also performed 2 sensitivity analyses. First, as the Swedish Patient Register only includes information from specialist care, we additionally considered use of prescribed psychotropic drugs as an indication of psychiatric disorder, through the Swedish Prescribed Drug Register, which includes data on all dispensed medications in Sweden since July 2005.^45^ Second, we restricted the analyses to participants born since January 1, 1987, to assess the impact of incomplete coverage of the Swedish Patient Register.

All analyses were conducted in SAS version 9.4 (SAS Institute) and R version 4.1 (R Foundation). A confidence interval that did not include 1 was considered statistically significant. Data were analyzed from March 2022 to April 2023.

## Results

The median (IQR) age at exposure to assault, injury, and bereavement was 22 (18-31; n = 49 957), 19 (8-40; n = 555 314), and 60 (51-68; n = 321 263), respectively (Table). Most individuals exposed to assault (35 091 [70%]) and injury (329 642 [59%]) were men, whereas 226 531 (71%) of those exposed to bereavement were women. During a median (IQR) follow-up of 4.9 (2.2-8.2), 9.1 (4.1-15.6), and 8.1 (3.4-14.8) years, the crude incidence rate of any psychiatric disorder was 38.1, 13.9, and 9.0 per 1000 person-years among individuals exposed to assault, injury, or bereavement, respectively.

**Table.** Characteristics of Study Cohorts for Exposure to Any Assault, Any Injury, or Bereavement

Characteristics	Any assault, No. (%)				Any injury, No. (%)				Any bereavement, No. (%)			
	Population-matched cohort		Sibling cohort		Population-matched cohort		Sibling cohort		Population-matched cohort		Sibling cohort	
	Exposed individuals (n = 49 957)	Unexposed individuals (n = 499 570)	Exposed individuals (n = 34 894)	Unexposed full siblings (n = 56 534)	Exposed individuals (n = 555 314)	Unexposed individuals (n = 555 3140)	Exposed individuals (n = 388 077)	Unexposed full siblings (n = 629 434)	Exposed individuals (n = 321 263)	Unexposed individuals (n = 321 2630)	Exposed individuals (n = 191 034)	Unexposed full siblings (n = 372 665)
Age at index date, median (IQR), y	22 (18-31)	22 (18-31)	22 (18-30)	23 (17-33)	19 (8-40)	19 (8-40)	19 (9-38)	22 (11-43)	60 (51-68)	60 (51-68)	57 (48-65)	55 (46-63)
Follow-up, median (IQR), y	4.9 (2.2-8.2)	5.6 (2.9-9.1)	5.1 (2.4-8.4)	5.7 (3-9.1)	9.1 (4.1-15.6)	9.3 (4.4-15.9)	9.5 (4.4-16.2)	9.8 (4.7-16.4)	8.1 (3.4-14.8)	7.7 (3.3-14.2)	8.6 (3.7-15.4)	8.9 (3.9-15.6)
No. of participants with incident psychiatric disorders (IR)	10 529 (38.1)	36 019 (11.6)	6779 (34.3)	5595 (15.9)	79 514 (13.9)	431 052 (7.3)	52 592 (12.7)	54 824 (8.0)	27 912 (9.0)	172 596 (5.8)	16 531 (8.6)	23 176 (6.1)
Sex												
Male	35 091 (70.2)	350 910 (70.2)	24 992 (71.6)	29 333 (51.9)	329 642 (59.4)	3 296 420 (59.4)	232 825 (60.0)	319 690 (50.8)	94 732 (29.5)	947 320 (29.5)	57 218 (30.0)	192 217 (51.6)
Female	14 866 (29.8)	148 660 (29.8)	9902 (28.4)	27 201 (48.1)	225 672 (40.6)	2 256 720 (40.6)	155 252 (40.0)	309 744 (49.2)	226 531 (70.5)	2 265 310 (70.5)	133 816 (70.0)	180 448 (48.4)
Educational level, y												
<9	776 (1.6)	5948 (1.2)	436 (1.2)	1078 (1.9)	24 939 (4.5)	243 406 (4.4)	15 050 (3.9)	32 202 (5.1)	79 365 (24.7)	709 785 (22.1)	41 714 (21.8)	81 723 (21.9)
9-12	37 427 (74.9)	302 934 (60.6)	26 094 (74.8)	37 196 (65.8)	310 996 (56.0)	2 849 500 (51.3)	222 148 (57.2)	352 546 (56.0)	172 683 (53.8)	1 618 272 (50.4)	105 664 (55.3)	208 496 (55.9)
>12	9512 (19.0)	169 347 (33.9)	7070 (20.3)	14 210 (25.1)	128 634 (23.2)	1 558 260 (28.1)	95 768 (24.7)	171 216 (27.2)	67 846 (21.1)	875 959 (27.3)	43 059 (22.5)	81 520 (21.9)
Unknown	2242 (4.5)	21 341 (4.3)	1294 (3.7)	4050 (7.2)	90 745 (16.3)	901 974 (16.2)	55 111 (14.2)	73 470 (11.7)	1369 (0.4)	8614 (0.3)	597 (0.3)	926 (0.2)
Annual family income level												
Lowest 20%	3784 (7.6)	24 669 (4.9)	2435 (7.0)	4320 (7.6)	26 140 (4.7)	251 691 (4.5)	17 493 (4.5)	29 379 (4.7)	903 (0.3)	12 188 (0.4)	499 (0.3)	1553 (0.4)
Middle	25 224 (50.5)	239 599 (48.0)	17 569 (50.3)	28 141 (49.8)	205 055 (36.9)	2 011 085 (36.2)	146 992 (37.9)	246 645 (39.2)	78 882 (24.6)	784 355 (24.4)	46 050 (24.1)	95 311 (25.6)
Top 20%	19 486 (39.0)	220 629 (44.2)	14 069 (40.3)	21 026 (37.2)	243 791 (43.9)	2 487 713 (44.8)	175 551 (45.2)	290 525 (46.2)	240 674 (74.9)	2 413 367 (75.1)	144 148 (75.5)	275 646 (74.0)
Unknown	1463 (2.9)	14 673 (2.9)	821 (2.4)	3047 (5.4)	80 328 (14.5)	802 651 (14.5)	48 041 (12.4)	62 885 (10.0)	804 (0.3)	2720 (0.1)	337 (0.2)	155 (0.04)
Marital status												
Single	43 057 (86.2)	418 953 (83.9)	30 313 (86.9)	45 685 (80.8)	430 675 (77.6)	4 277 630 (77.0)	304 352 (78.4)	461 827 (73.4)	61 660 (19.2)	756 122 (23.5)	41 644 (21.8)	110 065 (29.5)
Married or cohabiting	4013 (8.0)	66 285 (13.3)	2713 (7.8)	8486 (15.0)	90 856 (16.4)	1 010 893 (18.2)	62 500 (16.1)	133 447 (21.2)	195 143 (60.7)	1 937 696 (60.3)	111 774 (58.5)	209 201 (56.1)
Divorced or widowed	2887 (5.8)	14 332 (2.9)	1868 (5.4)	2363 (4.2)	33 783 (6.1)	264 617 (4.8)	21 225 (5.5)	34 160 (5.4)	64 460 (20.1)	518 812 (16.1)	37 616 (19.7)	53 399 (14.3)
Family history of psychiatric disorders^a^												
No	33 114 (66.3)	381 252 (76.3)	24 760 (71.0)	40 048 (70.8)	443 668 (79.9)	4 586 410 (82.6)	318 724 (82.1)	512 676 (81.5)	250 536 (78.0)	2 559 024 (79.7)	144 056 (75.4)	278 399 (74.7)
Yes	16 843 (33.7)	118 318 (23.7)	10 134 (29.0)	16 486 (29.2)	111 646 (20.1)	966 730 (17.4)	69 353 (17.9)	116 758 (18.5)	70 727 (22.0)	653 606 (20.3)	46 978 (24.6)	94 266 (25.3)
History of severe somatic diseases^b^												
No	45 018 (90.1)	461 228 (92.3)	31 635 (90.7)	51 731 (91.5)	498 558 (89.8)	5 135 937 (92.5)	352 248 (90.8)	582 228 (92.5)	254 696 (79.3)	2 586 467 (80.5)	157 099 (82.2)	312 732 (83.9)
Yes	4939 (9.9)	38 342 (7.7)	3259 (9.3)	4803 (8.5)	56 756 (10.2)	417 203 (7.5)	35 829 (9.2)	47 206 (7.5)	66 567 (20.7)	626 163 (19.5)	33 935 (17.8)	59 933 (16.1)
No. of trauma subtypes during follow-up												
1	48 516 (97.1)	NA	33 917 (97.2)	NA	486 785 (87.7)	NA	339 338 (87.4)	NA	320 053 (99.6)	NA	190 154 (99.5)	NA
2	1441 (2.9)	NA	977 (2.8)	NA	65 983 (11.9)	NA	47 040 (12.1)	NA	1210 (0.4)	NA	880 (0.5)	NA
≥3	NA	NA	NA	NA	2546 (0.5)	NA	1699 (0.4)	NA	NA	NA	NA	NA

Abbreviation: IR, crude incidence rate per 1000 person-years.

^a^ Marginal difference between exposed individuals and unexposed siblings is due to different numbers of siblings for exposed individuals. The family history of psychiatric disorders is always the same within each family.

^b^ Includes cardiovascular disease, chronic pulmonary disease, connective tissue disease, diabetes, kidney diseases, liver diseases, ulcer diseases, and HIV infection/AIDS.

**Figure 1.**
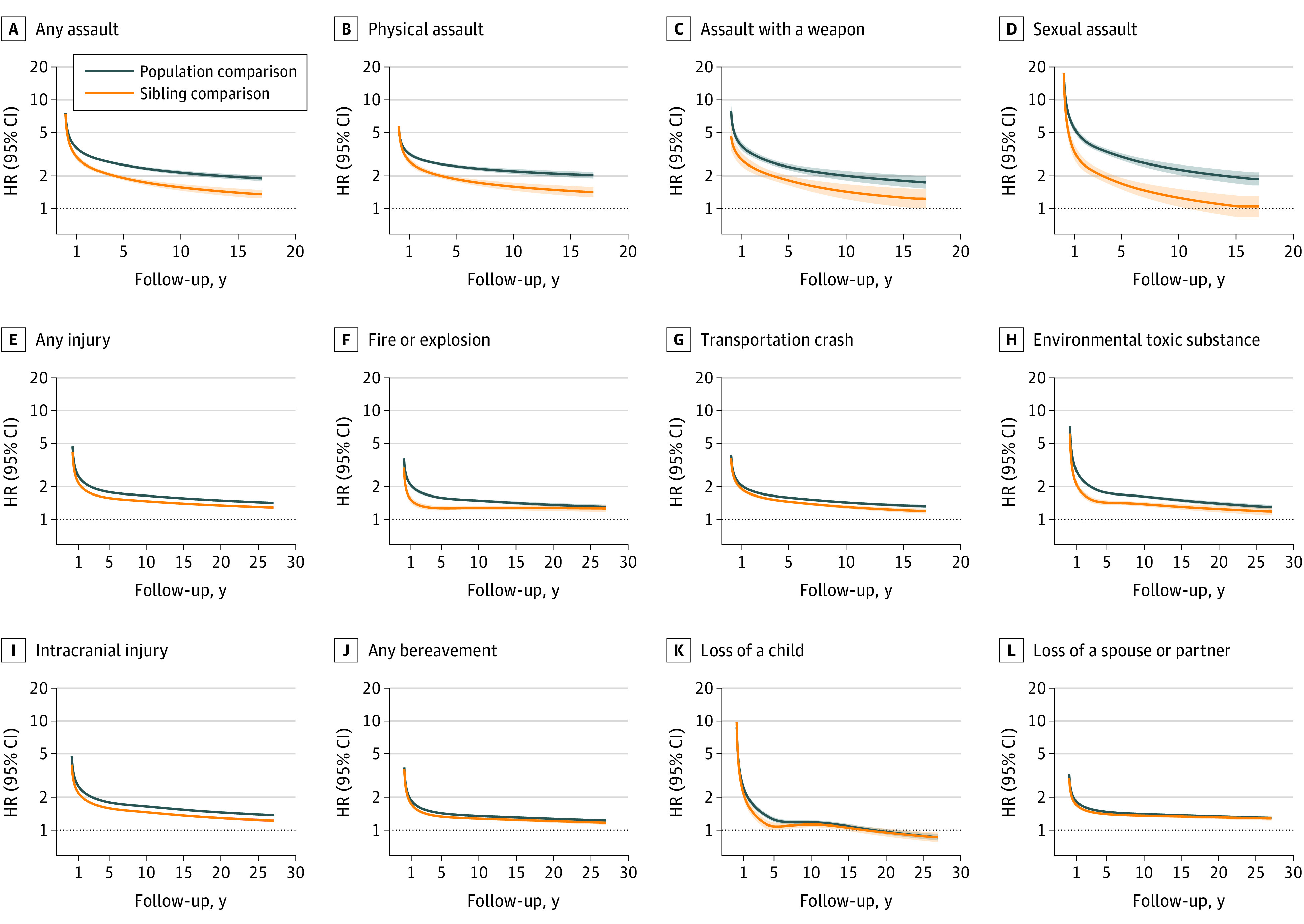
Time-Varying Hazard Ratios (HRs) of Any Psychiatric Disorder After Exposure to Potentially Traumatic Events HRs for any psychiatric disorder among individuals exposed to potentially traumatic events compared to matched unexposed individuals or unexposed full siblings from 1987 to 2013. Time-varying HRs and 95% CIs were calculated using flexible parametric survival models, allowing the effect of potentially traumatic events to vary over time. A spline with 4 *df* was used for the baseline hazard, while 2 *df* was used for the time-varying effect. Models were stratified by matching identifier (birth year, sex, and birthplace) for population comparison or family identifier for sibling comparison, controlling for age at index date, sex, educational level, family income, marital status, history of somatic diseases, and family history of psychiatric disorders (in population comparison). The longest follow-up time was 17 years for assaults or transportation incident, as data were available from 1997.

**Figure 2.**
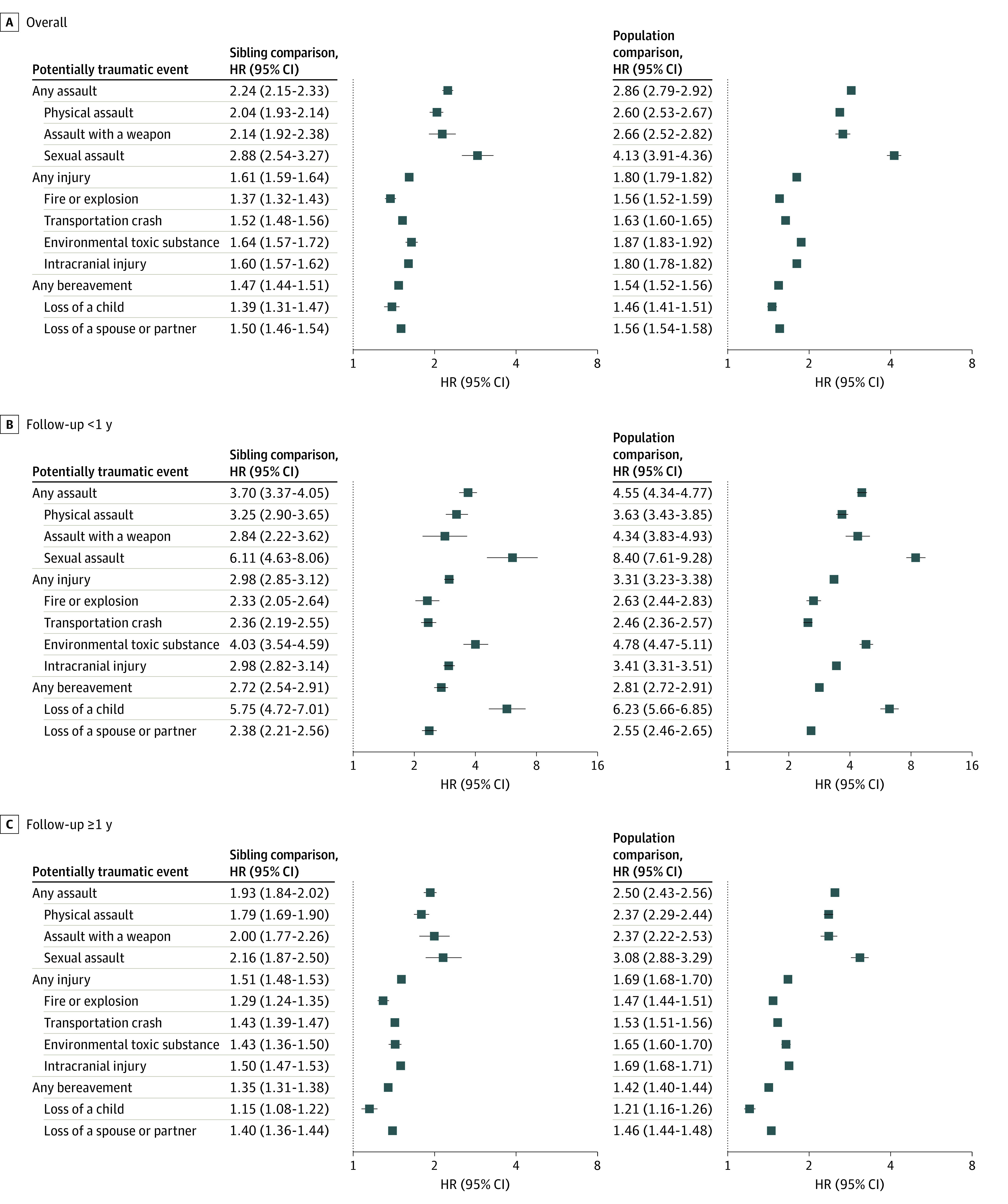
Hazard Ratios (HRs) of Any Psychiatric Disorder After Exposure to Potentially Traumatic Events by Follow-up Time HRs with 95% CIs for any psychiatric disorders among individuals exposed to potentially traumatic events compared to matched unexposed individuals or unexposed full siblings, overall and by time during follow-up (<1 and ≥1 year). Estimates were calculated from Cox models with time since the index date as the underlying time scale. Models were stratified by matching identifier (birth year, sex, and birthplace) in population comparison or family identifier in sibling comparison, controlling for age at index date, sex, educational level, family income, marital status, history of somatic diseases, and family history of psychiatric disorders (in population comparison).

The incidence rate of any psychiatric disorder increased considerably immediately after a potentially traumatic event, although the risk increment declined rapidly thereafter and remained somewhat stable beyond 1 year (Figure 1). During the first year of follow-up, the mean HR (95% CI) was 4.55 (4.34-4.77) for assault, 3.31 (3.23-3.38) for injury, and 2.81 (2.72-2.91) for bereavement (Figure 2). The corresponding HR (95% CI) beyond 1 year was 2.50 (2.43-2.56), 1.69 (1.68-1.70), and 1.42 (1.40-1.44), respectively. Comparable results were obtained in sibling comparison (HRs [95% CIs] during first year: assault, 3.70 [3.37-4.05], injury, 2.98 [2.85-3.12], and bereavement, 2.72 [2.54-2.91]; HRs after first year: assault, 1.93 [1.84-2.02], injury, 1.51 [1.48-1.53], and bereavement, 1.35 [1.31-1.38]. Different types of traumatic events were all associated with an increased rate of any psychiatric disorder in both population and sibling comparisons, with the greatest HRs noted during the first year after sexual assault, loss of a child, and injury with exposure to environmental toxic substance. After 1 year, the rate elevations were still present yet significantly attenuated.

**Figure 3.**
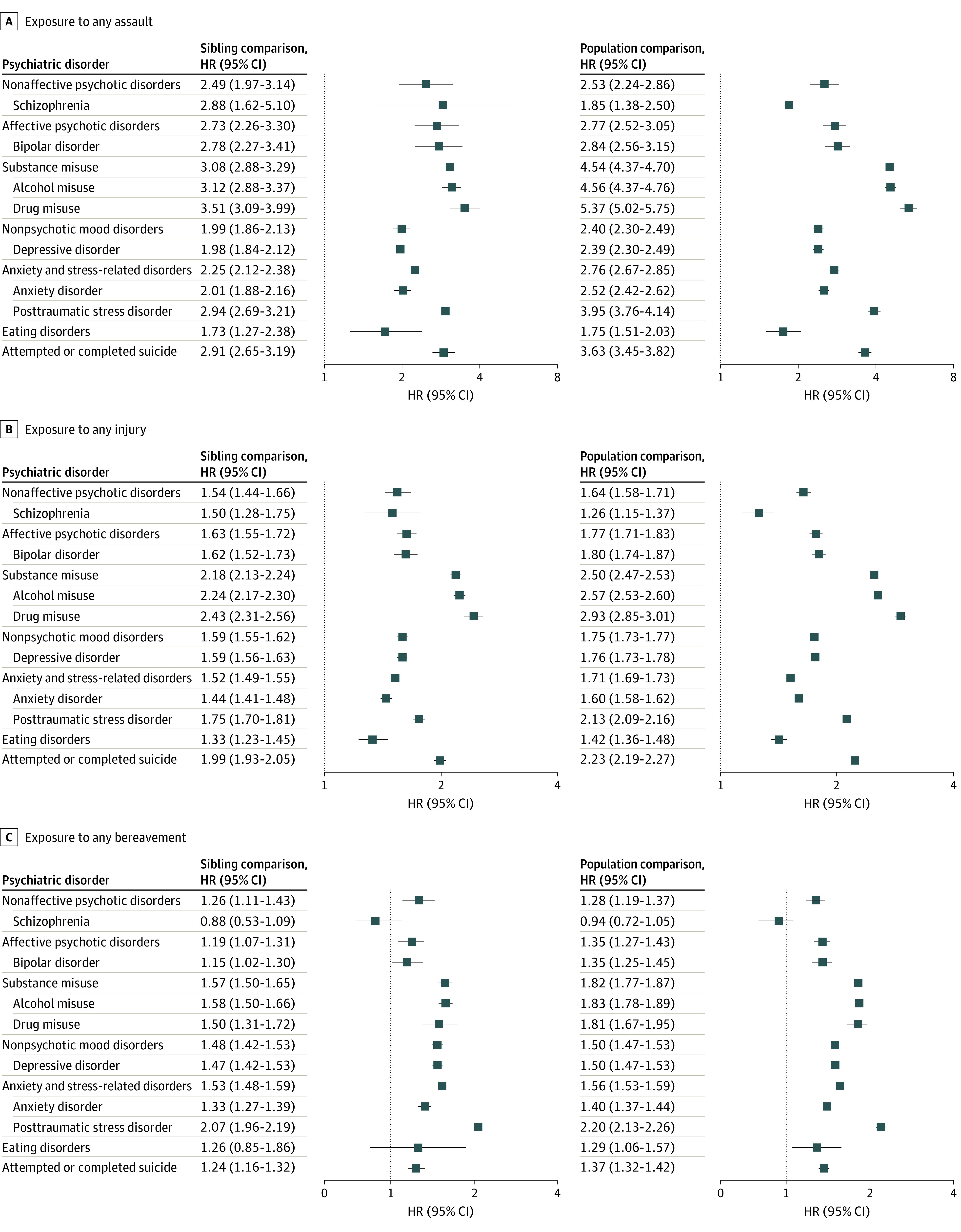
Hazard Ratios (HRs) of Various Types of Psychiatric Disorders After Exposure to Potentially Traumatic Events HRs with 95% CIs for categories and subtypes of psychiatric disorders among individuals exposed to potentially traumatic events compared to matched unexposed individuals or unexposed full siblings. Estimates were calculated from Cox models with time since the index date as the underlying time scale. Models were stratified by matching identifier (birth year, sex, and birthplace) in population comparison or family identifier in sibling comparison, controlling for age at index date, sex, educational level, family income, marital status, history of somatic diseases, and family history of psychiatric disorders (in population comparison).

**Figure 4.**
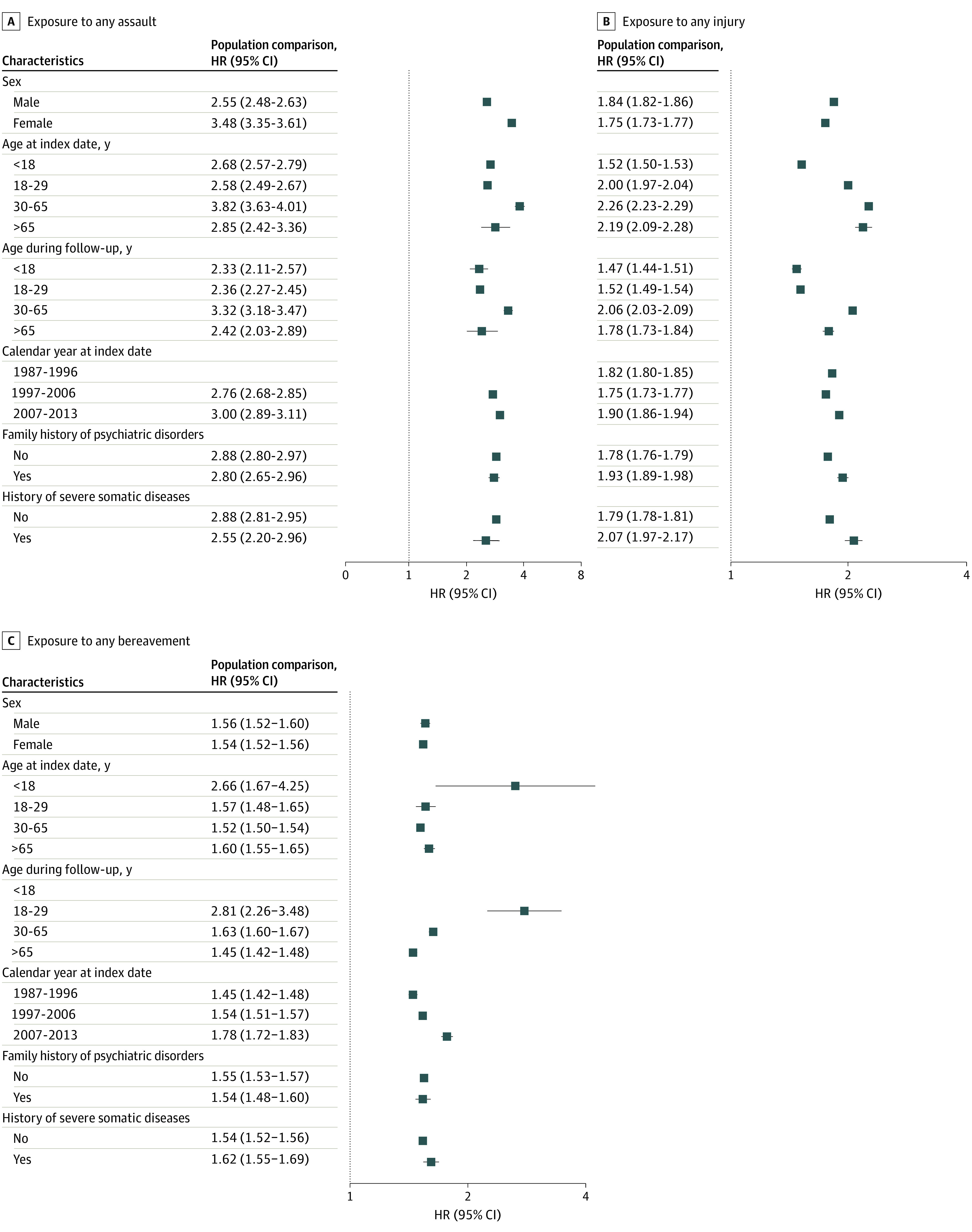
Hazard Ratios (HRs) of Any Psychiatric Disorder After Exposure to Potentially Traumatic Events, Stratified Analysis by Background Characteristics HRs with 95% CIs for psychiatric disorders among individuals exposed to potentially traumatic events compared to matched unexposed individuals, stratified by background characteristics. Estimates were calculated from Cox models with time since the index date as the underlying time scale. Models were stratified by matching identifier (birth year, sex, and birthplace), controlling for age at index date, sex, educational level, family income, marital status, history of somatic diseases, and family history of psychiatric disorders. Estimates are not available for any assault from 1987 to 1997 or for bereavement with age during follow-up younger than 18 years.

Most of the assessed potentially traumatic events were associated with the full range of assessed psychiatric disorders as well as attempted or completed suicides (Figure 3; eTable 2 in Supplement 1), except schizophrenia and eating disorders after bereavement. Sexual assault showed the strongest associations with all psychiatric disorders, especially with PTSD. We noted the strongest associations between assaults and injuries with drug misuse, with some attenuation in sibling estimates—particularly for assaults. In contrast, we found bereavement to be most strongly associated with PTSD with similar estimates obtained in population and sibling comparisons. The observed associations did not vary substantially by sex, age, calendar period, family history of psychiatric disorders, or history of severe somatic diseases (Figure 4; eFigure 2 in Supplement 1).

Within all cohorts, we observed greater risk increase of any psychiatric disorder among individuals exposed to multiple traumatic events than those exposed to only 1 traumatic event (eFigure 3 in Supplement 1). For example, in the sibling comparison, the HR was 3.44 (95% CI, 2.95-4.00) for exposure to 3 or more types of injury and 1.52 (95% CI, 1.50-1.55) for exposure to only 1 type of injury. We found similar associations when additionally using psychotropic medications as an indication of psychiatric disorders (eFigure 4 in Supplement 1) and by restricting the analyses to participants born since January 1, 1987 (eFigure 5 in Supplement 1).

## Discussion

To our knowledge, this is the first nationwide population-based, sibling-controlled study to provide comprehensive assessment of the association of potentially traumatic events with up to 27 years incidence trajectories of psychiatric disorders. Our data reveal that individuals exposed to potentially traumatic events, including assaults, injuries, and bereavement, were at an increased risk of almost all types of psychiatric disorders and that the risk increment was largely independent of familial background and history of severe somatic diseases. Of note, we did observe some influence of familial factors on incidence trajectories of psychiatric disorders after assaults, particularly in relation to substance misuse. The risk increment was generally observed during up to 27 years of follow-up but was most pronounced during the first year after the traumatic events, indicating a critical time window for intervention.

Our study corroborates the existing literature on an association between various traumatic events and psychiatric disorders. For instance, previous studies indicate that individuals exposed to childhood abuse,^46,47,48^ sexual violence,^8,14^ and traumatic brain injury^13,49^ are at increased risk of psychiatric disorders, including PTSD, depression, anxiety disorders, alcohol or drug misuse, and suicide attempt or self-harm. However, existing evidence rests primarily on descriptive^2^ or cross-sectional data,^14,23,46^ selected or small populations,^5,50^ or studies with lack of control for critical confounding factors,^4,14^ and familial factors are rarely controlled for.^13,51^ Similarly, increased risks of psychiatric disorders have frequently been reported after loss of a close relative without consideration of familial confounding.^3,10,11,52^ Using both population and full-sibling comparisons, we demonstrated associations of various potentially traumatic events with a wide range of psychiatric disorders, both in the short and long term, extending the existing knowledge base by showing limited role of familial confounding.

While the role of familial factors (eg, genetic factors^34,35^ and early-life environment^53,54,55^) in psychiatric disorders have been demonstrated, our findings indicate that familial factors may have limited influence on the occurrence of psychiatric disorders in the context of severe trauma or bereavement. We found that the associations of injury and bereavement with psychiatric disorders were minimally influenced by performing sibling-based comparisons (inherently controlling for familial factors), while the associations between assaults and psychiatric disorders (particularly substance misuse) were modestly attenuated in sibling comparisons vs population comparisons. These findings are in line with previous studies on psychiatric hospitalization following traumatic brain injuries using both population and sibling comparisons, suggesting a similarly limited impact of familial factors in the association between head trauma and psychiatric disorders.^13^ The noted attenuations in the association of assaults with psychiatric disorders in the sibling comparison may be due to genetic or early environmental factors (eg, upbringing) shared among siblings, predisposing the siblings to potential exposure to assaults as well as psychiatric disorders. Studies have indeed reported some overlap in familial liability between trauma exposure and PTSD symptoms.^32,56^ Nevertheless, the associations between assaults and psychiatric disorders remained significant in the sibling comparisons, indicating at most modest influence of familial factors. Thus, further studies on the role of familial factors in psychiatric disorders may benefit from incorporating data on major life events or trauma.

The time-varying risk pattern noted in our study is supported by previous studies on death of a close relative,^11,52^ natural disaster,^6^ and cancer diagnosis.^57^ Compared to prior studies, our study provides a somewhat more precise depiction of the time-varying nature of the link between trauma or bereavement and first psychiatric diagnoses. Overall, we observed a significantly increased risk of first psychiatric diagnosis immediately after the traumatic event or bereavement, and the risk elevation generally attenuated over time. This pattern was particularly distinct for sexual assault, injury with exposure to environmental toxic substances, and loss of a child, signaling the importance of early monitoring and interventions soon after these events. Nonetheless, more research is needed to determine for whom and when intervention is optimal, particularly after bereavement. Notably, after a dramatic rise in psychiatric disorders shortly after child loss, the rate elevation largely diminished beyond 5 years and disappeared beyond 15 years. This finding is in line with previous studies on psychiatric disorders after child loss^11,52^ and may be attributable to the extreme immediate psychiatric reaction to the devastating event of child loss as well as the fact that competing risks of death from other physical diseases^58^ may contribute to attenuated incidence rates of psychiatric disorders in this vulnerable population with time from the event.

In line with earlier reports,^4,23^ we found greater risk elevation of psychiatric disorders in relation to traumas involving interpersonal violence (ie, sexual and physical assault) than other trauma types. In contrast, our findings indicate that the risk increment of psychiatric disorders was more modest after bereavement, particularly following loss of a spouse or partner, compared to injuries or assaults, as previously indicated.^10,52^ Differences in age at exposure to bereavement, compared to assaults or injuries, may contribute to the varying increments in the incidence trajectories of psychiatric disorders. Similarly, differences in the expectedness of these events (ie, injuries and assaults are mostly sudden and unexpected while bereavement often comes with some degree of forewarning), may further contribute to these differences in relative risks. We found all trauma subtypes to be associated with a higher risk of most categories of psychiatric disorders, with the exception that bereavement was not associated with subsequent risks of schizophrenia and eating disorders—2 conditions with a strong genetic component^35^ and complex etiology. While we observed the strongest associations between sexual assaults and PTSD overall, assaults and injuries tended to be most strongly associated with substance misuse while bereavement demonstrated the strongest association with PTSD. While the underlying mechanisms for the variability in risk elevations across trauma to specific disorders awaits further study, we did observe some evidence for familial contribution to estimates after assaults, particularly in relation to substance misuse.

### Strengths and Limitations

The strengths of our study include the nationwide study population, which enabled detailed subgroup analyses by type of potentially traumatic events, category of psychiatric disorders, and participant characteristics. The prospectively and independently collected information on exposures and outcomes and the long follow-up time largely minimized the risk of selection and information biases. The inclusion of sibling comparison relieved largely the concerns of familial confounding.^36^

There are several limitations to acknowledge. First, incidence rates of psychiatric disorders are underestimated, as we have no data on patients who received their diagnosis in primary care only. Yet this concern was to some extent relieved by the virtually identical results in our sensitivity analyses when adding prescriptions of psychotropic medications as indication of cases without a specialist diagnosis of psychiatric disorders. We also missed less severe forms of traumatic events due to the sole reliance on diagnoses from inpatient and outpatient specialist care. This may have led to overestimation of the incidence rates of psychiatric disorders among the unexposed individuals, as some truly exposed individuals were misclassified as unexposed. This misclassification of the exposure (ie, trauma) is likely to result in underestimation of the presented associations. However, this does not apply to bereavement, where virtually all events are captured through the nationwide register resources. In contrast, it is possible that symptoms of normal grief early after bereavement are misdiagnosed as psychiatric disorders. However, the risk increments lasted for years after bereavement, suggesting a long-lasting psychiatric morbidity in this population. Since our study included only traumatic events and psychiatric disorders diagnosed through the hospital setting or specialized psychiatric clinics, our results are probably not generalizable to less severe traumatic events and psychiatric disorders treated outside hospital or clinic settings. In addition, individuals exposed to trauma or bereavement may be more closely monitored for psychiatric disorders, particularly early after these events. However, while this surveillance bias likely contributes to the short-term risk, the risk elevations lasted for over 20 years after these events, suggesting our findings are unlikely solely attributable to such bias. Additionally, carryover effects^59^ could be hypothesized a priori in sibling comparison, as unexposed siblings may also be affected by their sibling’s trauma, yielding potential underestimates in the sibling comparison. However, the incidence trajectories of psychiatric disorders after injuries and bereavement were almost identical in sibling and population comparisons, arguing against a general influence of such carryover effects on our results.

## Conclusions

Using a large Swedish population-based sibling-controlled cohort with over 20 years of follow-up, we found that individuals exposed to potentially traumatic events were at an increased subsequent risk of incident psychiatric disorders, largely independent of familial background. The greatest risk increase observed immediately after the events motivates enhanced clinical alertness of vulnerable individuals during this crucial time window.

## Notes

Supplement 1. eMethods. Ascertainment of potentially traumatic events, psychiatric disorders, and covariates eTable 1. International Classification of Diseases codes for identification of potentially traumatic events, psychiatric disorders, and covariates eTable 2. Hazard ratios of various psychiatric disorders after exposure to potentially traumatic events, based on sibling comparison eFigure 1. Study design eFigure 2. Hazard ratios of any psychiatric disorder after exposure to potentially traumatic events, stratified by characteristics, sibling-based comparison eFigure 3. Hazard ratios of any psychiatric disorder after exposure to multiple subtypes of potentially traumatic events eFigure 4. Hazard ratios of any psychiatric disorder after potentially traumatic events, adding prescriptions of psychotropic medications as an indication of psychiatric disorders eFigure 5. Hazard ratios of any psychiatric disorder after exposure to potentially traumatic events, restricted to participants born from 1987 onwards

Supplement 2. Data sharing statement
