# Supplementary material for: Incidence Trajectories of Psychiatric Disorders After Assault, Injury, and Bereavement
Source: JAMA Psychiatry. 2024 Jan 17;81(4):374–85. doi: 10.1001/jamapsychiatry.2023.5156 (PMC10794980; doi:10.1001/jamapsychiatry.2023.5156)
Supplement: Supplement 1. — eMethods. Ascertainment of potentially traumatic events, psychiatric disorders, and covariates eTable 1. International Classification of Diseases codes for identification of potentially traumatic events, psychiatric disorders, and covariates eTable 2. Hazard ratios of various psychiatric disorders after exposure to potentially traumatic events, based on sibling comparison eFigure 1. Study design eFigure 2. Hazard ratios of any psychiatric disorder after exposure to potentially traumatic events, stratified by characteristics, sibling-based comparison eFigure 3. Hazard ratios of any psychiatric disorder after exposure to multiple subtypes of potentially traumatic events eFigure 4. Hazard ratios of any psychiatric disorder after potentially traumatic events, adding prescriptions of psychotropic medications as an indication of psychiatric disorders eFigure 5. Hazard ratios of any psychiatric disorder after exposure to potentially traumatic events, restricted to participants born from 1987 onwards [file jamapsychiatry-e235156-s001.pdf]

Supplemental Online Content

Chen Y, Shen Q, Lichtenstein P, et al. Incidence trajectories of psychiatric disorders after assault, injury, and bereavement. *JAMA Psychiatry*. Published online January 17, 2024. doi:10.1001/jamapsychiatry.2023.5156

eMethods. Ascertainment of potentially traumatic events, psychiatric disorders, and covariates

eTable 1. International Classification of Diseases codes for identification of potentially traumatic events, psychiatric disorders, and covariates

eTable 2. Hazard ratios of various psychiatric disorders after exposure to potentially traumatic events, based on sibling comparison

eFigure 1. Study design

eFigure 2. Hazard ratios of any psychiatric disorder after exposure to potentially traumatic events, stratified by characteristics, sibling-based comparison

eFigure 3. Hazard ratios of any psychiatric disorder after exposure to multiple subtypes of potentially traumatic events

eFigure 4. Hazard ratios of any psychiatric disorder after potentially traumatic events, adding prescriptions of psychotropic medications as an indication of psychiatric disorders

eFigure 5. Hazard ratios of any psychiatric disorder after exposure to potentially traumatic events, restricted to participants born from 1987 onwards

This supplemental material has been provided by the authors to give readers additional information about their work.

© 2024 Chen Y et al. *JAMA Psychiatry.*

eMethods

Ascertainment of potentially traumatic events, psychiatric disorders, and covariates

Data sources

This study was based on the high-quality and nationwide population and health registers in Sweden. The Swedish Patient Register includes nationwide information on inpatient specialist care and on hospital-based outpatient specialist care.[1] Collection of inpatient care data started in 1964 (psychiatric care started in 1973 with initial coverage of 86%) and reached complete coverage since 1987. In Sweden it is mandatory for all physicians, private and publicly funded, to deliver data (including acute/emergency care, but not primary care data) to the Patient Register. Diagnoses in the Patient Register are coded according to the Swedish revisions of the International Classification of Disease (ICD) codes. Since 2001, it is obliged for public caregivers to report hospital-based outpatient hospital visits, including acute/emergency care visits (initial coverage >80%). The Causes of Death Register comprises virtually complete data on all deaths of Swedish residents since 1952.[2] The Multi-Generation Register includes largely complete familial linkage for individuals born since 1932 in Sweden.[3] The Prescribed Drug Register collects information on drugs redeemed with a prescription from all pharmacies in Sweden since July 2005.[4] The Longitudinal Integration Database for Health Insurance and Labor Market (LISA)[5] integrates information on marital status, employment, income, educational level, etc. on a yearly basis for Swedish residents aged ≥16 years since 1990. The Total Population Register[6] was also linked to identify demographic information (such as birth, sex) and to determine emigration.

Ascertainment of potentially traumatic events

Through the Swedish Patient Register, we used the Swedish revisions of the ICD-9 and ICD-10 codes to ascertain first recorded exposure to any assault (physical assault, assault with a weapon, or sexual assault) from 1997 to 2013 and injury (fire/explosion, transportation crash [available from 1997 to 2013], environmental toxic substance, or intracranial brain injury) from 1987 to 2013. Studies have demonstrated excellent validity on hospital discharge data on injuries in the Patient Register.[7,8] In terms of bereavement, we focused on bereavement due to loss of a child (<18 years old) or spouse/partner due to death. We used the Swedish Multi-Generation Register to identify children and, through a common child, spouse or partner of the study participants. We then linked the children and spouses/partners to the Swedish Causes of Death Register to identify bereavement events from 1987 to 2013. The corresponding ICD codes used to ascertain assaults and injuries are listed in **eTable 1**.

Ascertainment of psychiatric disorders

We retrieved first-onset psychiatric disorders (**eTable 1**) from the Swedish Patient Register, using both the primary and secondary diagnoses at the time of discharge. Validation studies for a range of specific psychiatric disorders[9-14] demonstrate high validity for these diagnoses in the Patient Register. We classified psychiatric disorders as non-affective psychotic disorders, affective psychotic disorders, substance misuse, non-psychotic mood disorders, anxiety, and stress-related disorders, and eating disorders. Suicide attempt and death from suicide were included as an additional outcome and were identified through the Patient Register and Causes of Death Register (**eTable 1**).

Covariates

We obtained information on the highest educational level, family income, and marital status collected during one year before the index date from the LISA database. Individuals under 16 years were assigned “unknown” status for education and income and assigned as “single” for marital status. History of severe somatic diseases, defined as having any of the following conditions before the index date: chronic pulmonary disease, connective tissue disease, diabetes, renal diseases, liver diseases, ulcer diseases, and HIV infection/AIDS, was derived from the Patient Register (**eTable 1**). Family history of psychiatric disorders, defined as having any diagnosed psychiatric disorder among biological parents or full siblings before the index date, was ascertained from the Patient Register.

References

1. Ludvigsson JF, Andersson E, Ekbom A, et al. External review and validation of the Swedish national inpatient register. *BMC Public Health*. Jun 9 2011;11:450. doi:10.1186/1471-2458-11-450
2. Brooke HL, Talback M, Hornblad J, et al. The Swedish cause of death register. *Eur J Epidemiol*. Sep 2017;32(9):765-773. doi:10.1007/s10654-017-0316-1
3. Ekbom A. The Swedish Multi-generation Register. *Methods Mol Biol*. 2011;675:215-20. doi:10.1007/978-1-59745-423-0_10
4. Wettermark B, Hammar N, Fored CM, et al. The new Swedish Prescribed Drug Register--opportunities for pharmacoepidemiological research and experience from the first six months. *Pharmacoepidemiol Drug Saf*. Jul 2007;16(7):726-35. doi:10.1002/pds.1294
5. Ludvigsson JF, Svedberg P, Olen O, Bruze G, Neovius M. The longitudinal integrated database for health insurance and labour market studies (LISA) and its use in medical research. *Eur J Epidemiol*. Apr 2019;34(4):423-437. doi:10.1007/s10654-019-00511-8
6. Ludvigsson JF, Almqvist C, Bonamy AK, et al. Registers of the Swedish total population and their use in medical research. *Eur J Epidemiol*. Feb 2016;31(2):125-36. doi:10.1007/s10654-016-0117-y
7. Gedeborg R, Engquist H, Berglund L, Michaelsson K. Identification of incident injuries in hospital discharge registers. *Epidemiology*. Nov 2008;19(6):860-7. doi:10.1097/ede.0b013e318181319e
8. Nilsson AC, Spetz CL, Carsjo K, Nightingale R, Smedby B. Reliability of the hospital registry. The diagnostic data are better than their reputation. *Lakartidningen*. 1994;91:603–605.
9. Kouppis E, Ekselius L. Validity of the personality disorder diagnosis in the Swedish National Patient Register. *Acta Psychiatr Scand*. May 2020;141(5):432-438. doi:10.1111/acps.13166
10. Kristjansson E, Allebeck P, Wistedt B. Validity of the diagnosis schizophrenia in a psychiatric inpatient register: A retrospective application of DSM-III criteria on ICD-8 diagnoses in Stockholm county. *Nordisk Psykiatrisk Tidsskrift*. 2009;41(3):229-234. doi:10.3109/08039488709103182
11. Sellgren C, Landen M, Lichtenstein P, Hultman CM, Langstrom N. Validity of bipolar disorder hospital discharge diagnoses: file review and multiple register linkage in Sweden. *Acta Psychiatr Scand*. Dec 2011;124(6):447-53. doi:10.1111/j.1600-0447.2011.01747.x
12. Birgegard A, Forsen Mantilla E, Dinkler L, et al. Validity of eating disorder diagnoses in the Swedish national patient register. *J Psychiatr Res*. Jun 2022;150:227-230. doi:10.1016/j.jpsychires.2022.03.064
13. Vilaplana-Perez A, Isung J, Krig S, et al. Validity and reliability of social anxiety disorder diagnoses in the Swedish National Patient Register. *BMC Psychiatry*. May 15 2020;20(1):242. doi:10.1186/s12888-020-02644-7
14. Hollander AC, Askegard K, Iddon-Escalante C, Holmes EA, Wicks S, Dalman C. Validation study of randomly selected cases of PTSD diagnoses identified in a Swedish regional database compared with medical records: is the validity sufficient for epidemiological research? *BMJ Open*. Dec 23 2019;9(12):e031964. doi:10.1136/bmjopen-2019-031964

© 2024 Chen Y et al. *JAMA Psychiatry.*

eTable 1. International Classification of Diseases codes for identification of potentially traumatic events, psychiatric disorders, and covariates

Trauma / Psychiatric disorder / Covariates	ICD-9	ICD-10	Data Source
**POTENTIALLY TRAUMATIC EVENTS**			
**Assault**			
Physical assault	E964, E969, E984, E987	X92, Y01-Y04, T741	National Patient Register
Assault with a weapon	E922, E965, E966, E985, E986	X93-Y00	National Patient Register
Sexual assault		T742, T762, Y05	National Patient Register
**Injury**			
Fire/explosive	940B, 940E, 940F, 940X, 941-949, E890-E899	X00-X08, T20-T25, T31	National Patient Register
Transportation crash	E807, E819, E826, E829, E838, E841, E849	V01-V99, Y85	National Patient Register (Inpatients only)
Environmental toxic substance	981-989	T52-T65	National Patient Register
Intracranial injury	800B, 800D, 801B, 801D, 803B, 803D, 85	S06	National Patient Register
**Bereavement**	**Definition**		
Loss of children	Loss of a child (< 18 years old)		Causes of Death Register, Multi-generation Register
Loss of spouse or partner	Loss of a spouse or partner (have a common biological offspring)		Causes of Death Register, Multi-generation Register
**PSYCHIATRIC DISORDERS**			
Any psychiatric disorder	291, 292, 295-311, 314, 317-319	F10-F69, F70-F73, F78, F79, F84, F90	National Patient Register
Non-affective psychotic disorders	295, 297, 298, excluding 295H and 298B	F20-F24, F28-F29	National Patient Register
Schizophrenia	295A-295E, 295G, 295W, 295X	F200-F206, F208, F209	National Patient Register
Affective psychotic disorders	296, 295H, 298B	F25, F30-F31, F323, F333	National Patient Register
Bipolar disorder	296A, 296C, 296D, 296E, 296W, 296X	F30, F31	National Patient Register
Substance misuse	291, 292, 303, 304	F10-F16, F18-F19	National Patient Register
Alcohol misuse	303A, 303X, 305A	F100-F109	National Patient Register
Drug misuse	304A-304H, 304W, 304X, 305X	F110-F119, F190-F199	National Patient Register
Non-psychotic mood disorders	300E, 311	F32-F34, excluding F323 and F333, F38-F39	National Patient Register
Depressive disorder	300E, 311	F32, F33, excluding F323 and F333	National Patient Register
Anxiety and stress-related disorders	300A-D, 300F-H, 300W, 300X, 306, 307A, 308, 309	F40-F48	National Patient Register
Anxiety disorder	300A, 300C	F400-F402, F408, F409, F410-F413, F418, F419	National Patient Register

© 2024 Chen Y et al. *JAMA Psychiatry.*

Post-traumatic stress disorder	308A-308E, 308X, 309A-309E, 309W, 309X	F430-F432, F438, F439	National Patient Register
Eating disorders	307B, 307F	F500, F501, F502, F503, F509	National Patient Register
Attempted/completed suicide	E950-E959, E980-E989	X60-X84, Y10-Y34	National Patient Register, Death of cause Register
**COVARIATES**			
**History of severe somatic diseases**			
Cardiovascular disease	390-438, 440, 444, 445	I00-I70, I730, I74-I75	National Patient Register
Chronic pulmonary disease	490-496	J40-J46	National Patient Register
Connective tissue disease	710A, 710B, 710E, 714A, 714B, 714C, 714W, 714X, 725	M05, M06, M32-M34, M351, M353	National Patient Register
Diabetes	250	E10-E14	National Patient Register
Renal diseases	582, 583	N01, N03, N052-N057	National Patient Register
Liver diseases	571C, 571E, 571F, 571G, 572C, 572D, 572E, 572W, 456A, 456B, 456C	K702-K704, K717, K721, K729, K73, K74, K766, K767, I850, I859, I982, I983	National Patient Register
Ulcer diseases	531-534	K25-K28	National Patient Register
HIV infection/AIDS	042-044	B20-B24	National Patient Register

ICD: International Classification of Diseases.

© 2024 Chen Y et al. *JAMA Psychiatry.*

eTable 2. Hazard ratios of psychiatric disorders after exposure to various potentially traumatic events, based on sibling comparison

	HR (95% CI) for types of psychiatric disorders by types of potentially traumatic events [a]								
**Potentially traumatic events**	**PTSD**	**Anxiety disorder**	**Alcohol misuse**	**Drug misuse**	**Depressive disorder**	**Bipolar disorder**	**Schizophrenia**	**Eating disorders**	**Attempted and death from suicide**
Physical assault	2.63 (2.34-2.95)	1.83 (1.67-2.00)	2.89 (2.63-3.18)	3.22 (2.75-3.76)	1.92 (1.75-2.10)	2.22 (1.72-2.87)	2.19 (1.11-4.31)	1.18 (0.74-1.89)	2.65 (2.35-2.97)
Assault with a weapon	2.52 (2.00-3.18)	1.92 (1.57-2.33)	2.69 (2.20-3.29)	2.44 (1.82-3.27)	1.81 (1.50-2.19)	4.01 (2.08-7.74)	NA	4.38 (0.90-21.2)	2.93 (2.30-3.75)
Sexual assault	4.52 (3.56-5.73)	2.57 (2.13-3.11)	2.87 (2.24-3.68)	3.54 (2.25-5.57)	1.95 (1.61-2.37)	4.02 (2.36-6.83)	2.53 (0.40-15.9)	1.93 (1.16-3.22)	3.15 (2.38-4.17)
Fire/explosion	1.48 (1.35-1.62)	1.24 (1.15-1.34)	1.50 (1.38-1.63)	1.71 (1.47-2.00)	1.42 (1.32-1.52)	1.54 (1.27-1.87)	1.25 (0.75-2.07)	1.29 (0.98-1.69)	1.69 (1.55-1.85)
Transportation crash	1.75 (1.65-1.85)	1.42 (1.36-1.49)	1.75 (1.66-1.85)	2.15 (1.96-2.37)	1.53 (1.47-1.60)	1.44 (1.28-1.62)	1.50 (1.04-2.15)	1.25 (1.06-1.47)	1.99 (1.88-2.10)
Environmental toxic substance	1.89 (1.71-2.08)	1.61 (1.49-1.75)	2.02 (1.84-2.21)	2.29 (1.97-2.66)	1.58 (1.46-1.70)	1.65 (1.34-2.02)	1.30 (0.80-2.11)	1.40 (1.04-1.89)	1.74 (1.58-1.92)
Intracranial injury	1.67 (1.61-1.73)	1.41 (1.37-1.45)	2.24 (2.17-2.32)	2.22 (2.09-2.35)	1.57 (1.52-1.61)	1.63 (1.52-1.75)	1.46 (1.22-1.74)	1.39 (1.26-1.53)	1.80 (1.74-1.86)
Loss of a child	2.29 (2.07-2.54)	1.10 (1.00-1.23)	1.14 (1.00-1.29)	1.29 (1.00-1.66)	1.17 (1.06-1.28)	1.03 (0.81-1.31)	0.78 (0.35-1.71)	0.90 (0.49-1.68)	1.13 (0.99-1.29)
Loss of a spouse or partner	2.00 (1.87-2.13)	1.38 (1.31-1.46)	1.69 (1.60-1.79)	1.63 (1.39-1.91)	1.56 (1.50-1.63)	1.23 (1.06-1.42)	0.80 (0.54-1.19)	1.62 (0.94-2.79)	1.29 (1.21-1.39)

Abbreviations: HR, hazard ratio; CI, confidence interval; NA, not available, due to rare outcomes.

[a] Estimates were derived from Cox models in sibling-based comparison, stratified by family identifier and adjusted for age, sex, educational level, family income, marital status, and history of somatic diseases.

© 2024 Chen Y et al. *JAMA Psychiatry.*

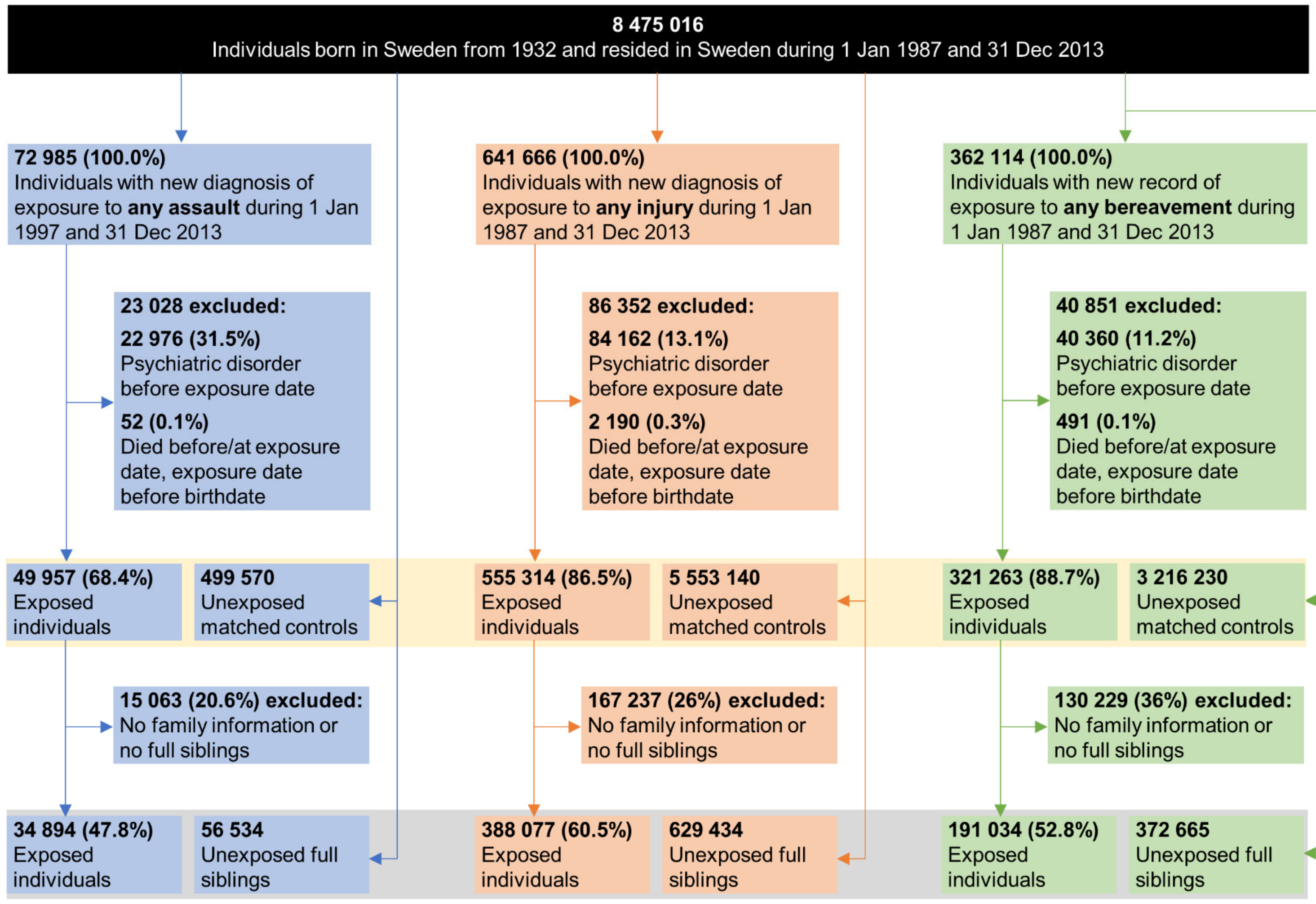

eFigure 1. Study design

Individuals were identified from the Swedish National Patient Register (Inpatient/Outpatient), Causes of Death Register, and Multi-Generation Register. The three individual panels (in blue, brown, and green) indicate the selection of study cohorts for exposure to any assault (including physical assault, assault with a weapon, or sexual assault), any injury (including fire/explosion, transportation crash, environmental toxic substances, or intracranial injury), and any bereavement (due to death of a child or spouse/partner), respectively. Comparisons based on population-matched cohorts and sibling-control cohorts are shown with yellow and grey backgrounds, respectively.

Unexposed individuals in the population-matched cohorts (1:10 individually matched by sex and birth year, and birthplace) were randomly selected from general population. The matching was performed using the density sampling method. Unexposed individuals and unaffected siblings were free of the interested exposure and free of psychiatric disorders before index date (study entry for unexposed individuals or unaffected siblings).

© 2024 Chen Y et al. *JAMA Psychiatry.*

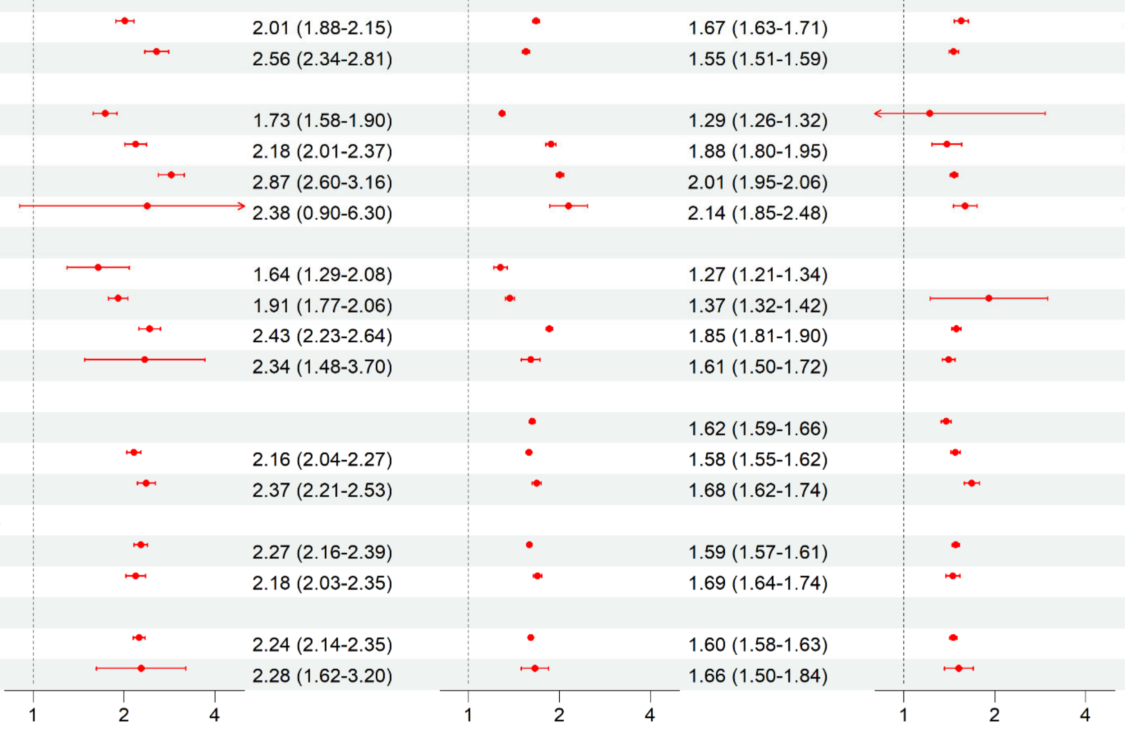

Characteristics	Exposure to any assault: HR (95% CI) Sibling	Exposure to any injury: HR (95% CI) Sibling	Exposure to any bereavement: HR (95% CI) Sibling
**Sex**			
Male	2.01 (1.88-2.15)	1.67 (1.63-1.71)	1.55 (1.47-1.63)
Female	2.56 (2.34-2.81)	1.55 (1.51-1.59)	1.46 (1.41-1.51)
**Age at index date, years**			
< 18	1.73 (1.58-1.90)	1.29 (1.26-1.32)	1.22 (0.51-2.93)
18 - 29	2.18 (2.01-2.37)	1.88 (1.80-1.95)	1.39 (1.24-1.55)
30 - 65	2.87 (2.60-3.16)	2.01 (1.95-2.06)	1.47 (1.43-1.51)
> 65	2.38 (0.90-6.30)	2.14 (1.85-2.48)	1.60 (1.46-1.74)
**Age during follow-up, years**			
< 18	1.64 (1.29-2.08)	1.27 (1.21-1.34)	
18 - 29	1.91 (1.77-2.06)	1.37 (1.32-1.42)	1.91 (1.22-2.99)
30 - 65	2.43 (2.23-2.64)	1.85 (1.81-1.90)	1.49 (1.44-1.55)
> 65	2.34 (1.48-3.70)	1.61 (1.50-1.72)	1.41 (1.34-1.47)
**Calendar year at index date**			
1987-1996		1.62 (1.59-1.66)	1.38 (1.33-1.43)
1997-2006	2.16 (2.04-2.27)	1.58 (1.55-1.62)	1.48 (1.43-1.53)
2007-2013	2.37 (2.21-2.53)	1.68 (1.62-1.74)	1.68 (1.59-1.78)
**Family history of psychiatric disorders**			
No	2.27 (2.16-2.39)	1.59 (1.57-1.61)	1.48 (1.44-1.53)
Yes	2.18 (2.03-2.35)	1.69 (1.64-1.74)	1.45 (1.38-1.53)
**History of severe somatic diseases**			
No	2.24 (2.14-2.35)	1.60 (1.58-1.63)	1.46 (1.42-1.50)
Yes	2.28 (1.62-3.20)	1.66 (1.50-1.84)	1.52 (1.36-1.69)

eFigure 2. Hazard ratios of any psychiatric disorder after exposure to potentially traumatic events, stratified by characteristics, sibling-based comparison.

Hazard ratios (HRs) with 95% confidence intervals (CIs) for psychiatric disorders among individuals exposed to potentially traumatic events, compared to unexposed full siblings, stratified analyses by background characteristics. Estimates were calculated from Cox models with time since the index date as underlying time scale. Models were stratified by family identifier, controlling for age at index date, sex, educational level, family income, marital status, and history of somatic diseases. Estimates are not available for any assault during 1987-1997, and for bereavement with age during follow-up <18.

© 2024 Chen Y et al. *JAMA Psychiatry.*

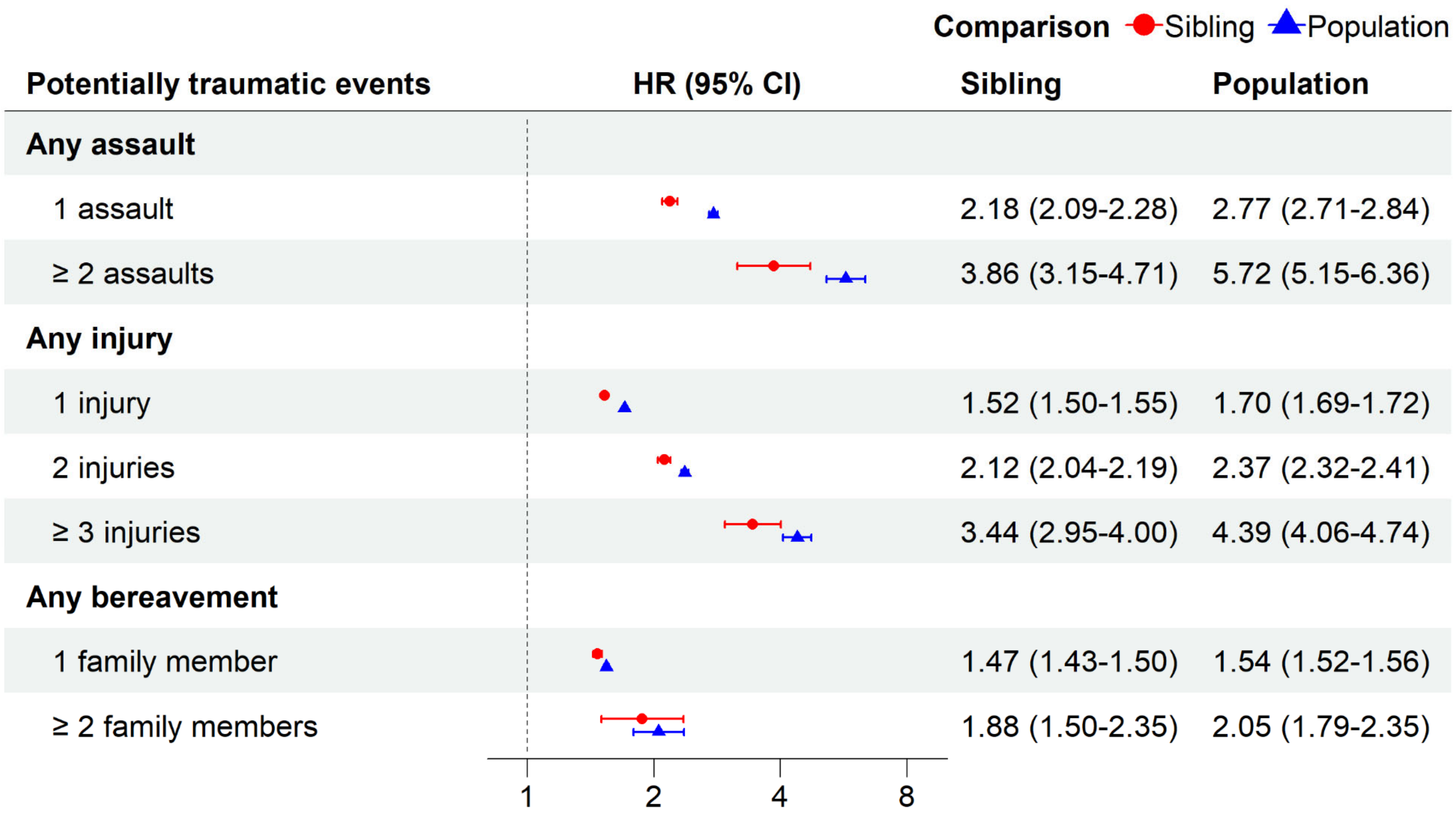

Potentially traumatic events	Sibling	Population
**Any assault**		
1 assault	2.18 (2.09-2.28)	2.77 (2.71-2.84)
≥ 2 assaults	3.86 (3.15-4.71)	5.72 (5.15-6.36)
**Any injury**		
1 injury	1.52 (1.50-1.55)	1.70 (1.69-1.72)
2 injuries	2.12 (2.04-2.19)	2.37 (2.32-2.41)
≥ 3 injuries	3.44 (2.95-4.00)	4.39 (4.06-4.74)
**Any bereavement**		
1 family member	1.47 (1.43-1.50)	1.54 (1.52-1.56)
≥ 2 family members	1.88 (1.50-2.35)	2.05 (1.79-2.35)

eFigure 3. Hazard ratios of any psychiatric disorder after exposure to multiple subtypes of potentially traumatic events.
Hazard ratios (HRs) with 95% confidence intervals (CIs) for psychiatric disorders among individuals exposed to multiple subtypes of potentially traumatic events, compared to matched unexposed individuals or unexposed full siblings. Estimates were calculated from Cox models with time since the index date as underlying time scale. Models were stratified by matching identifier (birth year, sex, and birthplace) in population comparison or family identifier in sibling comparison, controlling for age at index date, sex, educational level, family income, marital status, history of somatic diseases, and family history of psychiatric disorders (in population comparison).

© 2024 Chen Y et al. *JAMA Psychiatry.*

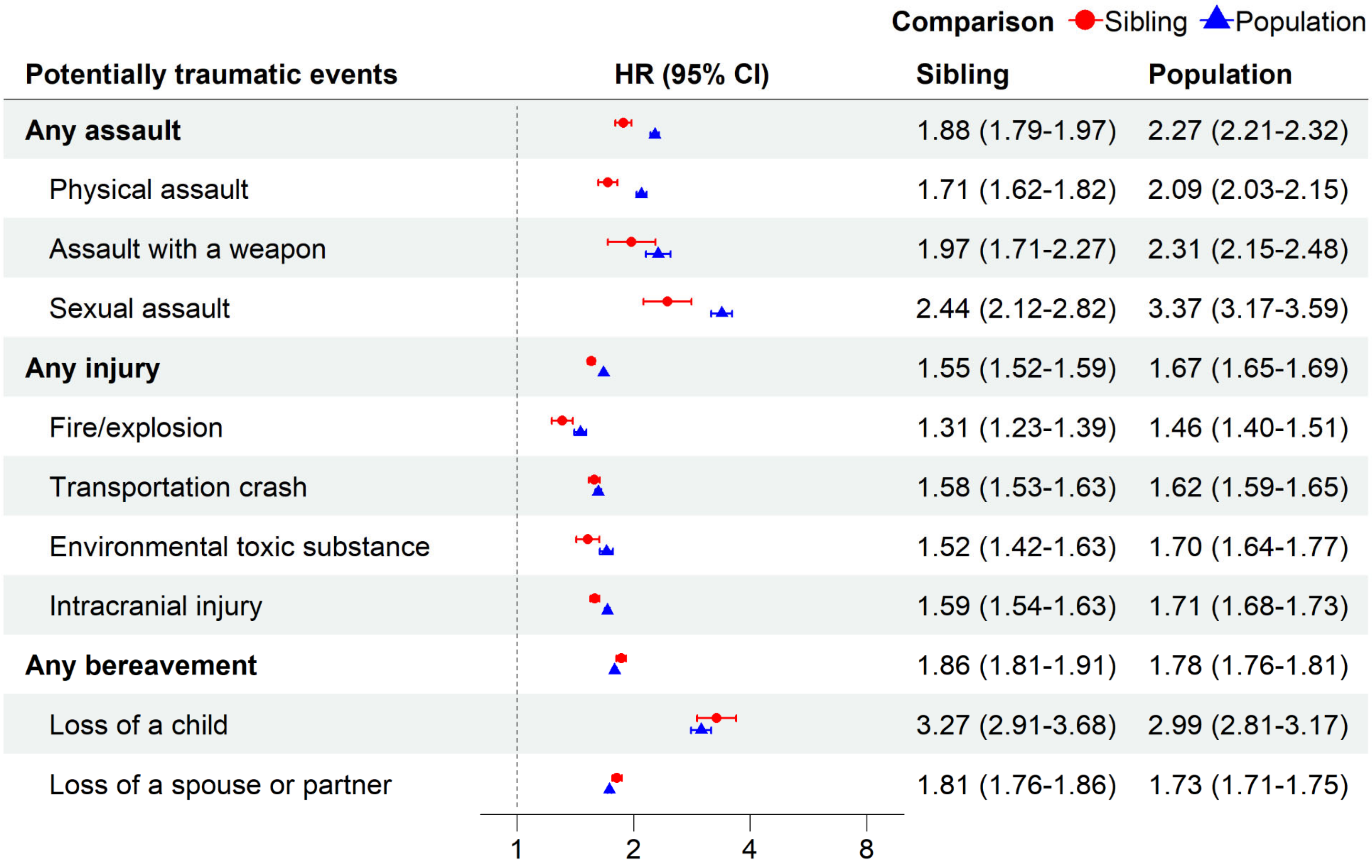

eFigure 4. Hazard ratios of any psychiatric disorder after potentially traumatic events, adding prescriptions of psychotropic medications as an indication of psychiatric disorders.

This analysis was restricted to the study period from 2005 to 2013 because the Prescribed Drug Register collects information since 2005. HR, hazard ratio; CI, confidence interval. Estimates were calculated from Cox models with time since the index date as underlying time scale. Models were stratified by matching identifier (birth year, sex, and birthplace) in population comparison or family identifier in sibling comparison, controlling for age at index date, sex, educational level, family income, marital status, history of somatic diseases, and family history of psychiatric disorders (in population comparison).

© 2024 Chen Y et al. *JAMA Psychiatry.*

Potentially traumatic events	Sibling	Population
**Any assault**	1.99 (1.85-2.14)	2.71 (2.62-2.81)
Physical assault	1.69 (1.54-1.86)	2.34 (2.24-2.44)
Assault with a weapon	2.14 (1.68-2.72)	2.36 (2.11-2.64)
Sexual assault	2.54 (2.12-3.04)	3.82 (3.56-4.10)
**Any injury**	1.30 (1.26-1.33)	1.49 (1.47-1.51)
Fire/explosion	1.13 (1.05-1.23)	1.30 (1.25-1.36)
Transportation crash	1.28 (1.22-1.35)	1.44 (1.40-1.48)
Environmental toxic substance	1.39 (1.29-1.50)	1.57 (1.51-1.63)
Intracranial injury	1.27 (1.23-1.31)	1.47 (1.45-1.50)
**Any bereavement**	2.26 (0.98-5.25)	4.21 (2.96-5.97)
Loss of a child	1.86 (0.71-4.87)	4.99 (3.25-7.66)
Loss of a spouse or partner	8.28 (1.33-93.10)	5.43 (2.65-11.13)

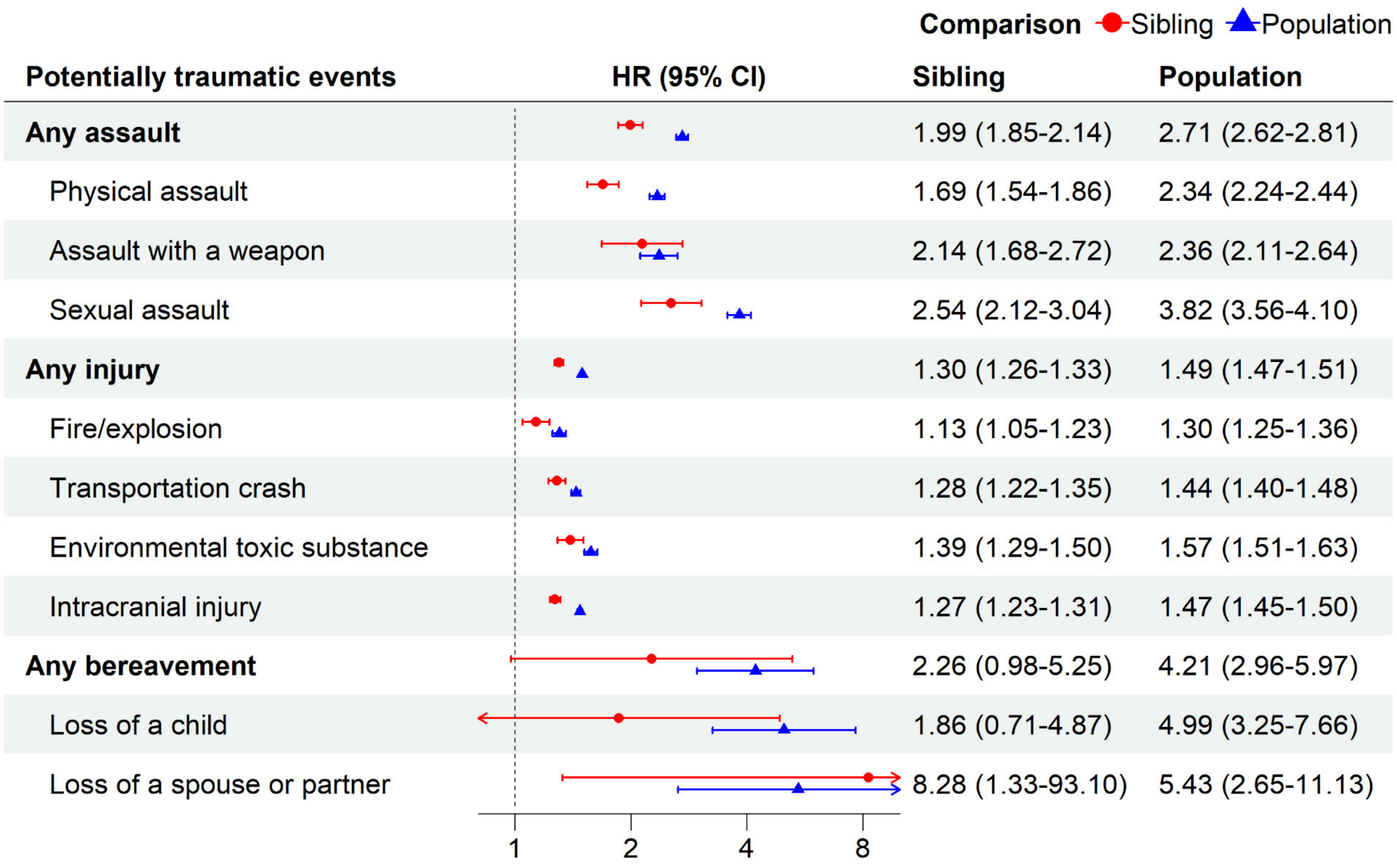

eFigure 5. Hazard ratios of any psychiatric disorder after exposure to potentially traumatic events, restricted to participants born from 1987 onwards.

HR, hazard ratio; CI, confidence interval. Estimates were calculated from Cox models with time since the index date as underlying time scale. Models were stratified by matching identifier (birth year, sex, and birthplace) in population comparison or family identifier in sibling comparison, controlling for age at index date, sex, educational level, family income, marital status, history of somatic diseases, and family history of psychiatric disorders (in population comparison).

© 2024 Chen Y et al. *JAMA Psychiatry.*
