# Supplementary material for: Incidence Trajectories of Psychiatric Disorders After Assault, Injury, and Bereavement
Source: JAMA Psychiatry. 2024 Jan 17;81(4):374–85. doi: 10.1001/jamapsychiatry.2023.5156 (PMC10794980; doi:10.1001/jamapsychiatry.2023.5156)
Supplement: Supplement 2. — Data sharing statement [file jamapsychiatry-e235156-s002.pdf]

Data Sharing Statement

Chen. Incidence Trajectories of Psychiatric Disorders After Assault, Injury, and Bereavement. *JAMA Psychiatry*. Published January 17, 2024. doi:10.1001/jamapsychiatry.2023.5156

Data
Data available: No

Additional Information
Explanation for why data not available: The register-based data cannot be made publicly available by the investigators but can be requested (subject to IRB approval) from the National Board of Health and Welfare.
